# Benchmarking specialty hospitals, a scoping review on theory and practice

**DOI:** 10.1186/s12913-017-2154-y

**Published:** 2017-04-04

**Authors:** A. Wind, W. H. van Harten

**Affiliations:** 1 Department of Psychosocial Research and Epidemiology, The Netherlands Cancer Institute-Antoni van Leeuwenhoek Hospital, Amsterdam, The Netherlands; 2 Department of Health Technology and Services Research, University of Twente, P.O. Box 217, 7500 AE Enschede, The Netherlands; 3 CEO Rijnstate Hospital, Arnhem, The Netherlands

**Keywords:** Benchmarking, Specialty hospitals, Quality improvement

## Abstract

**Background:**

Although benchmarking may improve hospital processes, research on this subject is limited. The aim of this study was to provide an overview of publications on benchmarking in specialty hospitals and a description of study characteristics.

**Methods:**

We searched PubMed and EMBASE for articles published in English in the last 10 years. Eligible articles described a project stating benchmarking as its objective and involving a specialty hospital or specific patient category; or those dealing with the methodology or evaluation of benchmarking.

**Results:**

Of 1,817 articles identified in total, 24 were included in the study. Articles were categorized into: pathway benchmarking, institutional benchmarking, articles on benchmark methodology or -evaluation and benchmarking using a patient registry. There was a large degree of variability:(1) study designs were mostly descriptive and retrospective; (2) not all studies generated and showed data in sufficient detail; and (3) there was variety in whether a benchmarking model was just described or if quality improvement as a consequence of the benchmark was reported upon. Most of the studies that described a benchmark model described the use of benchmarking partners from the same industry category, sometimes from all over the world.

**Conclusions:**

Benchmarking seems to be more developed in eye hospitals, emergency departments and oncology specialty hospitals. Some studies showed promising improvement effects. However, the majority of the articles lacked a structured design, and did not report on benchmark outcomes. In order to evaluate the effectiveness of benchmarking to improve quality in specialty hospitals, robust and structured designs are needed including a follow up to check whether the benchmark study has led to improvements.

**Electronic supplementary material:**

The online version of this article (doi:10.1186/s12913-017-2154-y) contains supplementary material, which is available to authorized users.

## Background

Healthcare institutions are pressured by payers, patients and society to deliver high-quality care and have to strive for continuous improvement. Healthcare service provision is becoming more complex, leading to quality and performance challenges [1]. In addition, there is a call for transparency on relative performance between and within healthcare organizations [2]. This pushes providers to focus on performance and show the added value for customers/patients [3, 4].

Without objective data on the current situation and comparison with peers and best practices, organizations cannot determine whether their efforts are satisfactory or exceptional, and specifically, what needs improvement. Benchmarking is a common and effective method for measuring and analyzing performance. The Joint commission defines benchmarking as:

*A systematic*, *data*-*driven process of continuous improvement that involves internally and*/*or externally comparing performance to identify*, *achieve*, *and sustain best practice. It requires measuring and evaluating data to establish a target performance level or benchmark to evaluate current performance and comparing these benchmarks or performance metrics with similar data compiled by other organizations*, *including best*-*practice facilities* [5].

Benchmarking may improve hospital processes, though according to Van Lent et al. [6], benchmarking as a tool to improve quality in hospitals is not well described and possibly not well developed. Identifying meaningful measures that are able to capture the quality of care in its different dimensions remains a challenging aspiration [7].

Before embarking on an international project to develop and pilot a benchmarking tool for quality assessment of comprehensive cancer care (the BENCH-CAN project [8]) there was a need to establish the state of the art in this field, amongst others to avoid duplication of work. The BENCHCAN project [8] aims at benchmarking comprehensive cancer care and yield good practice examples at European Cancer Centers in order to contribute to improvement of multidisciplinary patient treatment. This international benchmark project included 8 pilot sites from three geographical regions in Europe (North-West (*N* = 2), South (*N* = 3), Central-East (*N* = 3). The benchmarking study was executed according to the 13 steps developed by van Lent et al. [6], these steps included amongst others the construction of a framework, the development of relevant and comparable indicators selected by the stakeholders and the measuring and analysing of the set of indicators. Accordingly, we wanted to obtain an overview on benchmarking of specialty hospitals and specialty care pathways. Schneider et al. [9] describe specialty hospitals as hospitals “that treat patients with specific medical conditions or those in need of specific medical or surgical procedures” (pp.531). These are standalone, single-specialty facilities.

The number of specialty hospitals is increasing [9]. Porter [10] suggests that specialization of hospitals improves performance; it results in a better process organization, improved patient satisfaction, increased cost-effectiveness and better outcomes. According to van Lent et al. [6] specialty hospitals represent a trend, however, the opinions about the added value are divided. More insight into the benchmarking process in specialty hospitals could be useful to study differences in organization and performance and the identification of optimal work procedures [6]. Although specialty hospitals may differ according to discipline they have similarities such as the focus on one disease category and the ambition to perform in sufficient volumes. The scope of the BENCH-CAN [8] project was on cancer centers and cancer pathways, however, we did not expect to find sufficient material on this specific categories and thus decided to focus on specialty hospitals in general. Against this background, we conducted a scoping review. A scoping review approach provides a methodology for determining the state of the evidence on a topic that is especially appropriate when investigating abstract, emerging, or diverse topics, and for exploring or mapping the literature [11] which is the goal of this study. This study had the following objectives: (i) provide an overview of research on benchmarking in specialty hospitals and care pathways, (ii) describe study characteristics such as method, setting, models/frameworks, and outcomes, (iii) verify the quality of benchmarking as a tool to improve quality in specialty hospitals and identify success factors.

## Method

### Scoping systematic review

There are different types of research reviews which vary in their ontological, epistemological, ideological, and theoretical stance, their research paradigm, and the issues that they aim to address [12]. Scoping reviews have been described as a process of mapping the existing literature or evidence base. Scoping studies differ from systematic reviews in that they provide a map or a snapshot of the existing literature without quality assessment or extensive data synthesis [12]. Scoping studies also differ from narrative reviews in that the scoping process requires analytical reinterpretation of the literature [11]. We used the framework as proposed by Arksey and O’Mally [13]. This framework consist of 6 steps: (i) identifying the research question, (iii) study selection, (iv) charting the data, (v) collating, summarizing and reporting the results, (vi) optional consultation. Step 6 (optional consultation) was ensured by asking stakeholders from the BENCH-CAN project for input. Scoping reviews are a valuable resource that can be of use to researchers, policy-makers and practitioners, reducing duplication of effort and guiding future research.

### Data sources and search methods

**Fig. 1:**
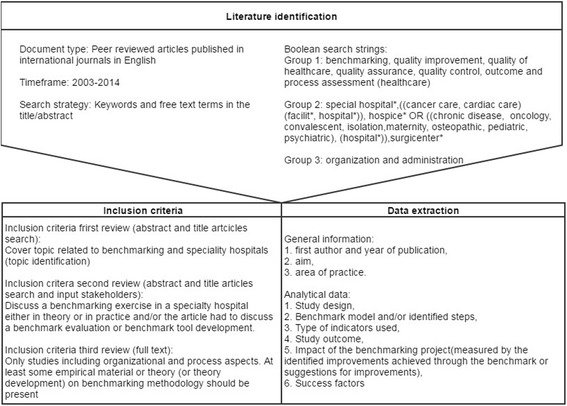
Research design

We performed searches in Pubmed and EMBASE. To identify the relevant literature, we focused on peer-reviewed articles published in international journals in English between 2003 and 2014. According to Saggese et al. [14] “this is standard practice in bibliometric studies, since these sources are considered ‘certified knowledge’ and enhance the results’ reliability” (pp.4). We conducted Boolean searches using truncated combinations of three groups of keywords and free text terms in title/abstract (see Fig. 1). The first consists of keywords concerning benchmarking and quality control. The second group includes key words regarding type of hospitals. All terms were combined with group 3: organization and administration. Different combinations of keywords led to different results, therefore five different searches in PubMed and four in EMBASE were performed. The full search strategies are presented in the Additional file 1. To retrieve other relevant publications, reference lists of the selected papers were used for snowballing. In addition stakeholders involved in the BENCH-CAN project [8] were asked to provide relevant literature.

### Selection method/article inclusion and exclusion criteria

Using abstracts, we started by excluding all articles that clearly did not meet the inclusion criteria, which covered topics not related to benchmarking and specialty hospitals. The two authors independently reviewed the remaining abstracts and made a selection using the following criteria: The article had to discuss a benchmarking exercise in a specialty hospital either in theory or in practice and/or the article had to discuss a benchmark evaluation or benchmark tool development. Only studies including organizational and process aspects were used, so studies purely benchmarking clinical indicators were excluded. At least some empirical material or theory (or theory development) on benchmarking methodology should be present; essays mainly describing the potential or added value of benchmarking without proving empirical evidence were thus excluded. The articles also had to appear in a peer-reviewed journal. The full texts were reviewed and processed by the first author. Only papers written in English were included.

### Data extraction

General information was extracted in order to be able to provide an overview of research on benchmarking in specialty hospitals and care pathways. The following information was extracted from the included articles: first author and year of publication, aim, and area of practice. The analytical data were chosen according to our review objective. They included the following: (I) study design, (II) Benchmark model and/or identified steps, (III) type of indicators used, (IV) Study outcome, (V) The impact of the benchmarking project (measured by the identified improvements achieved through the benchmark or suggestions for improvements), and (VI) Success factors identified. The first author independently extracted the data and the second author checked 25% of the studies to determine inter-rater reliability.

### Classification scheme benchmark models

At present, there is no standard methodology to classify benchmark models within healthcare in general and more specifically within specialty hospitals and care pathways. Therefore we looked at benchmark classification schemes outside the healthcare sector, especially in industry. A review of benchmarking literature showed that there are different types of benchmarking and a plethora of benchmarking process models [15]. One of these schemes was developed by Fong et al. [16] (Table 1). This scheme gives a clear description of each element included in the scheme and will therefore be used to classify the benchmark models described in this paper. It can be used to assess academic/research-based models. These models are developed mainly by academics and researchers mainly through their own research, knowledge and experience (this approach seems most used within the healthcare sector). This differs from Consultant/expert-based models (developed from personal opinion and judgment through experience in providing consultancy to organizations embarking on a benchmarking project) and Organization-based models (models developed or proposed by organizations based on their own experience and knowledge. They tend to be highly dissimilar, as each organization is different in terms of its business scope, market, products, process, etc.) [16].

**Table 1:** Classification scheme for benchmarking by Fong et al. [16]

Classification	Type	Meaning
Nature of benchmarking partner	Internal	Comparing within one organization about the performance of similar business units or processes
	Competitor	Comparing with direct competitors, catch up or even surpass their overall performance
	Industry	Comparing with company in the same industry, including noncompetitors
	Generic	Comparing with an organization which extends beyond industry boundaries
	Global	Comparing with an organization where its geographical location extends beyond country
Content of benchmarking	Process	Pertaining to discrete work processes and operating systems
	Functional	Application of the process benchmarking that compares particular business functions at two or more organizations
	Performance	Concerning outcome characteristics, quantifiable in terms of price, speed, reliability, etc.
	Strategic	Involving assessment of strategic rather than operational matters
Purpose for the relationship	Competitive	Comparison for gaining superiority over others
	Collaborative	Comparison for developing a learning atmosphere and sharing of knowledge

## Results

### Review

**Fig. 2:**
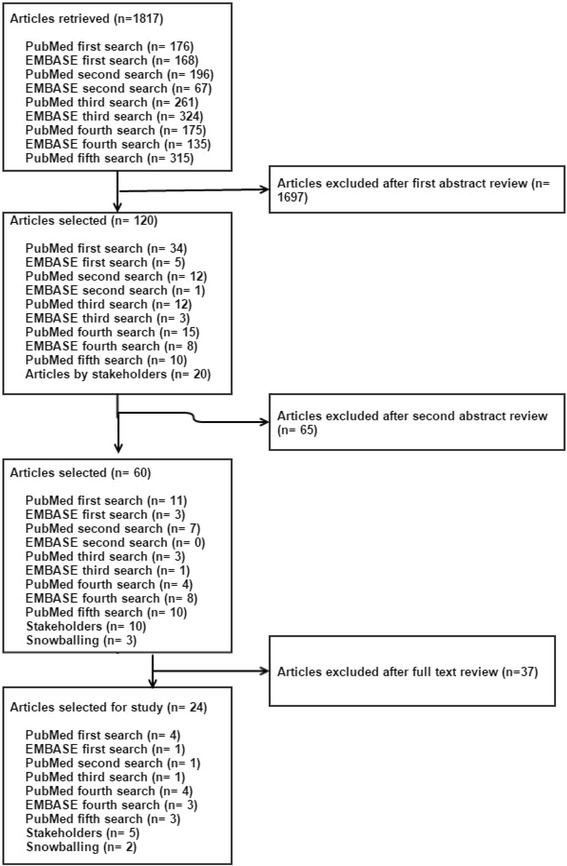
Article selection process

The search strategy identified 1,817 articles. The first author applied the first review eligibility criteria, the topic identification (Fig. 1), to the titles and abstracts. After this initial examination 1,697 articles were excluded. Two authors independently reviewed the abstracts of 120 articles. Snowballing identified three new articles that were not already identified in the literature search. Sixty articles were potentially eligible for full text review. The full text of these 60 publications were reviewed by two authors, resulting in a selection of 24 publications that met all eligibility criteria (see Figs. 1 and 2).

### Study characteristics

**Fig. 3:**
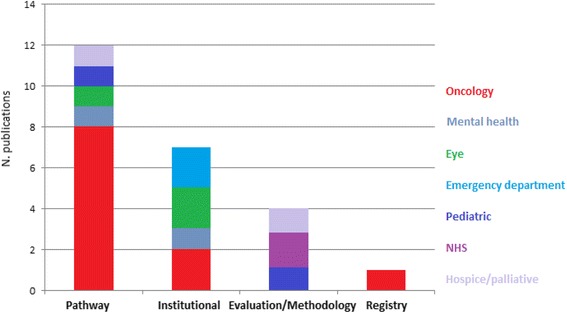
Number of publications per category and area of practice

Table 2 provides an overview of the general information of the included articles. To assist in the analysis, articles were categorized into: pathway benchmarking, institutional benchmarking, benchmark evaluation/methodology and benchmarking using a patient registry (see Fig. 3). For each category the following aspects will be discussed: study design, benchmark model and/or identified steps, type of indicators used, Study outcome, impact of the benchmarking project (improvements/improvement suggestions) and success factors. The benchmark model and/or described steps will be classified using the model by Fong [16].

**Table 2:** Charting categories and associated content for the general information on the benchmarking studies

First author (Year)	Aim	Area of practice
Brucker (2008) [27]	Establish a nationwide network of breast centres; to define suitable quality indicators (QIs) for benchmarking the quality of breast cancer (BC) care; to demonstrate existing differences in BC care quality; and to show that BC care quality improved with benchmarking from 2003 to 2007.	Breast cancer centers Germany
Chung (2010) [17]	Developing organization-based core measures for colorectal cancer patient care and apply these measures to compare hospital performance.	Hospitals registered in the TCDB program in Taiwan
Hermann (2006) [18]	To identify quality measures for international benchmarking of mental healthcare that assess important processes and outcomes of care, are scientifically sound, and are feasible to construct from pre-existing data.	Mental health care professionals from six countries (UK, Sweden, Canada, Australia, Denmark, and the USA) and one international organization, the European Society for Quality in Healthcare (ESQH)
Mainz (2009) [19]	Describing and analyzing the quality of care for important diseases in the Nordic countries (Denmark, Finland, Greenland, Iceland, Norway and Sweden).	Cancer treatment facilities from the different Nordic countries (Denmark, Finland, Greenland, Iceland, Norway and Sweden)
Miransky (2003) [20]	Describing the development of a database for benchmarking outcomes for cancer patients.	A consortium of 12 Comprehensive Cancer Centers in the US
Roberts (2012) [28]	The study had three main aims, to: (i) adapt the acuity-quality workforce planning method used extensively in the UK National Health Service (NHS) for use in hospices; (ii) compare hospice and NHS palliative care staffing establishments and their implications; and (iii) create ward staffing benchmarks and formulae for hospice managers.	Twenty-three palliative care and hospice wards, geographically representing England.
Setoguchi (2008) [24]	Comparing prospectively and retrospectively defined benchmarks for the quality of end-of-life care, including a novel indicator for the use of opiate analgesia.	Seniors with breast, colorectal, lung, or prostate cancer who participated in state pharmaceutical benefit programs in New Jersey and Pennsylvania
Stewart (2007) [21]	Develop tools that lead to better-informed decision making regarding practice management and physician deployment in comprehensive cancer centers and determine benchmarks of productivity using RVUs (Relative value units) accrued by physicians at each institution.	13 major academic cancer institutions with membership or shared membership in the National Comprehensive Cancer Network (NCCN)
Stolar (2010) [22]	Performing a blinded confidential financial performance survey of similar university pediatric surgery sections to start benchmarking performance and define relationships.	19 pediatric surgery sections of university children’s hospitals
Van Vliet (2010) [23]	Comparing process designs of three high-volume cataract pathways in a lean thinking framework and to explore how efficiency in terms of lead times, hospital visits and costs is related to process design.	Three eye hospitals in the UK, the USA and the Netherlands
Wallwiener (2011) [25]	Summarize the rationale for the creation of breast centres and discus the studies conducted in Germany. To obtain proof of principle for a voluntary, external benchmarking programme and proof of concept for third-party dual certification of breast centres and their mandatory quality management systems.	Breast centers in Germany
Wesselman (2014) [26]	Present data from the third annual analysis of the DKG-certified colorectal cancer centers with a particular focus on indicators for colorectal cancer surgery.	Colorectal cancer centers certified by the German Cancer Society (DKG)
Barr (2012) [30]	Revision of 2011 predictions with the use of National Practice Benchmark (NPB) reports from 2011 and development of new predictions. Design of a conceptual framework for contemplating these data based on an ecological model of the oncology delivery system.	Oncology practices in the USA
Brann (2011) [31]	The performance of child and adolescent mental health organizations. To provide an overview of the findings from two projects, undertaken to explore the variability in organizations’ performances on particular KPIs (key performance indicators).	Six child and adolescent mental health organizations
De Korne (2010) [3]	The purpose of this study was to evaluate the applicability of an international benchmarking initiative in eye hospitals.	Nine eye hospitals spread over Asia (3), Australia (1), Europe (4), and North America (1).
De Korne (2012) [29]	The aims of this study were to assess the applicability of a benchmarking project in U.S. eye hospitals and compare the results with an international initiative.	Five eye hospitals in the US
Schwappach (2003) [32]	Assess the effects of uniform indicator measurement and group benchmarking. This was followed by hospital-specific activities on clinical performance measures and patients’ experiences with emergency care in Switzerland.	Emergency departments of 12 community hospitals in Switzerland, participating in the ‘Emerge’ project.
Shaw (2003) [33]	To answer basic questions, using precise definitions, regarding emergency department (ED) utilization, wait times, services, and attending physician staffing of representative pediatric EDs (PEDs).	21 Pediatric emergency departments (PED) from 14 states of the USA.
Van Lent (2010) [6]	Examine benchmarking as part of an approach to improve performance in specialty hospitals	International comprehensive cancer centres (CCC) or departments within a CCC in Europe and the US
Ellershaw (2008) [34]	To evaluate the utility of participating in two benchmarking exercises to assess the care delivered to patients in the dying phase using the Liverpool Care Pathway for the Dying Patient (LCP).	Two cancer networks in the northwest of England
Ellis (2006) [35]	Review published descriptions of benchmarking activity and synthesize benchmarking principles to encourage the acceptance and use of Essence of Care as a new approach to continuous quality improvement, and to promote its acceptance as an integral and effective part of benchmarking activity in health services.	NHS (UK)
Matykiewicz (2005) [36]	Introduce Essence of Care, a benchmarking tool for health care practitioners and an integral part of the UK National Health Service (NHS) Clinical Governance agenda	Health care practitioners NHS (UK)
Profit (2010) [37]	To present a conceptual framework to develop comprehensive, robust, and transparent composite indicators of pediatric care quality, and to highlight aspects specific to quality measurement in children.	The Pediatric Data Quality Systems (Pedi-QS) Collaborative Measures Workgroup (consensus panel by the National Association of Children’s Hospitals and Related Institutions, Child Health Corporation of America, and Medical Management Planning)
Greene (2009) [38]	Describing The Role of the Hospital Registry in Achieving Outcome Benchmarks in Cancer Care	Carolinas Medical Center (US)

### I Pathway benchmarking (PB)

A summary analysis of the pathway benchmarking studies can be found in Table 3.

**Table 3:** Summary of the analysis of the pathway benchmarking projects

Author	Study design	Benchmarking model and/or steps	Indicators	Outcomes	Impact (improvements/improvement suggestions)	Success factors
Brucker [27]	Prospective interventional multi-centre feasibility study.	Partner: Industry Content: Performance Purpose: Collaborative Independent, scientific benchmarking system. Nine guideline-based quality targets serving as rate-based QIs (Quality Indicators) were initially defined, reviewed annually and modified or expanded accordingly. QI changes over time were analyzed descriptively	Quality outcome indicators derived from clinically relevant parameters.	The results from this study provide proof of concept for the feasibility of a novel, voluntary, nationwide system for benchmarking the quality of BC care	Marked QI (Quality Indicators) increases indicate improved quality of BC care.	The project was voluntary and all data was anonymized.
Chung [17]	Multi comparisons study and the development of core measures for colorectal cancer including a modified Delphi method.	N.A.	Quantitative structure, process and outcome indicators	Developing core measures for cancer care was a first step to achieving standardized measures for external monitoring, as well as for providing feedback and serving as benchmarks for cancer care quality improvement.	N.A.	N.A.
Hermann [18]	Multi comparisons study and indicator consensus development process (with elements of the Delphi method).	Partner: Industry/Global Content: Performance/Process Purpose: Collaborative Development of indicators for benchmarking.	Process and outcome indicators.	The bench mark was not performed, indicators were developed for a possible benchmark.	N.A.	N.A.
Mainz [19]	Multi comparisons study and the development of indicators based on consensus of a working group	N.A. The results that are available for the prioritized quality indicators cannot really be used for true comparisons and benchmarking	Outcome indicators	A major difference between the Nordic countries has been identified with regard for 5 years survival for prostate cancer.	N.A.	N.A.
Miransky [20]	Multi comparisons study with stakeholder consensus methods. Use of a specialized database for benchmarking outcomes for cancer patients. Conference calls and joint meetings between comprehensive cancer centers and possible benchmark vendors were used to develop this benchmarking database.	Partner: Industry Content: Performance Purpose: Collaborative	Development of a database containing outcome indicators. Benchmarking clinical outcomes and patient	The various databases developed by the collaborative provided the tools through which the group accomplished its goals.	Each consortium member is expected to participate in one quality improvement initiative annually	N.A.
Roberts [28]	Multi comparisons study on staffing and inpatient data at hospices. Study design drew extensively from a UK-wide nursing study (The UK Best practice Nursing Database).	N.A.	Mixture of indicators, both qualitative and quantitative and process and outcome indicators	A broader NHS ward data system, was successfully converted for hospice use. The resultant hospice and palliative care ward data show that, compared to NHS palliative care wards, charitable hospices: (i) look after fewer patients, but generate greater workloads owing to higher patient-dependency and acuity scores; (ii) are much better staffed; and (iii) achieve higher service-quality scores.	N.A.	N.A.
Setoguchi [24]	Retrospective and prospective cohort study.	Partner: Industry Content: Performance Purpose: Collaborative Defined benchmark measures for the quality of end-of-life cancer care previously developed by Earle et al. New measures were defined for the use of opiate analgesia, which included the proportion of patients who received an outpatient prescription for a long-acting opiate; a short-acting or a long-acting opiate; or both a short acting and a long-acting opiate.	Outcome indicators	Retrospective and prospective measures, including a new measure of the use of opiate analgesia, identified similar physician and hospital patterns of end-of-life care.	Findings suggest that the use of opiates at the end of life can be improved	N.A.
Stewart [21]	Multi comparisons study (clinical productivity and other characteristics of oncology physicians). Data collection by survey	Partner: Industry Content: Performance Purpose: Collaborative Established productivity benchmarks. The clinical productivity and other characteristics were reviewed of oncology physicians practicing in 13 major academic cancer institutions.	Outcome productivity indicators	Specific clinical productivity targets for academic oncologists were identified. A methodology for analyzing potential factors associated with clinical productivity and developing clinical productivity targets specific for physicians with a mix of research, administrative, teaching, and clinical salary support.	N.A.	N.A.
Stolar [22]	Multi comparisons study using a non-searchable anonymous data capture form through SurveyMonkey. Feedback from stakeholders and availability of information was used to develop indicators. A final questionnaire, containing 17 questions, was send to thirty pediatric surgery practices.	N.A.	Quantitative outcome indicators	A review of the clinical revenue performance of the practice illustrates that pediatric surgeons are unable to generate sufficient direct financial resources to support their employment and practice operational expenses.	The value of the services must accrue to a second party	N.A.
Van Vliet [23]	A retrospective comparative benchmark study with a mixed-method design	Partner: Industry/Global Content: Strategic Purpose: Collaborative The method comprised of 6 steps: (1) operational focus; (2) autonomous work cell; (3) physical lay-out of resources; (4) multi-skilled team; (5) pull planning and (6) elimination of wastes.	N/A	The environmental context and operational focus primarily influenced process design of the cataract pathways.	When pressed to further optimize their processes, hospitals can use these systematic benchmarking data to decrease the frequency of hospital visits, lead times and costs.	N.A.
Wallwiener [25]	Review of existing literature/data.	Partner: Industry Content: Performance Purpose: Collaborative Phase 1: Benchmarking; Phase 1a: proof of principle: Develop quality indicators; Phase 1b: analysis for a single specific specialty: to demonstrate the feasibility of subgroup analysis. Phase 2: certification of breast centres: to implement a quality management system to assess structural, process and outcome quality. Phase 3: nationwide implementation of certified breast centres.	Structural and process indicators	The voluntary benchmarking programme has gained wide acceptance among DKG/DGS-certified breast centres. The goal of establishing a nationwide network of certified breast centres in Germany can be considered largely achieved.	Improvements in surrogate parameters as represented by structural and process quality indicators suggest that outcome quality is improving.	N.A.
Wesselman [26]	Review of existing literature/data. Analysis of existing benchmarking reports of cancer centers.	Partner: Industry Content: Performance Purpose: Collaborative Analysis of benchmarking reports by the certified centers with the OnkoZert data which reflects the centers’ reference results over a period of 3 years. The data for these reports are collected by the centers using an electronic questionnaire and are submitted to OnkoZert. (an independent institute that organizes the auditing procedure on behalf of the DKG)	Respective and guideline-based outcome indicators	The present analysis of the results, together with the centers’ statements and the auditors’ reports, shows that most of the targets for indicator figures are being better met over the course of time.	There is a clear potential for improvement and the centers are verifiably addressing this.	N.A.

N.A. = Not applicable

### PB Study design

Study design varied across the different pathway studies. Most studies (*N* = 7) [17–22] used multiple comparisons, from which five studies sought to develop indicators. Different methods were used for this indicator development such as a consensus method (Delphi) [17–19]. In other articles a less structured way of reaching consensus was used such as conference calls [20] and surveys [21]. One study used a prospective interventional design [14] while another study [23] used a retrospective comparative benchmark study with a mixed-method design. Setoguchi et al. [24] used a combination of prospective and retrospective designs. Existing literature was used in two studies [25, 26]. More information on study design can be found in Table 3.

### PB Benchmark model

Eight articles described a benchmarking model and/or benchmarking steps. Applying the classification scheme by Fong et al. [16] most studies used benchmarking partners from the same industry (*N* = 6) [20, 21, 24–27]. Two studies also used partners from the industry but on the global level. A total of 6 studies benchmarked performance [20, 24–27], one study benchmarked performance and processes [18] and another study used strategic benchmarking [23]. All studies used benchmarking for collaborative purposes. For more information about the benchmark models see Table 3.

### PB Indicators

Most of the pathway studies used outcome indicators (*N* = 7) [19–22, 24, 26, 27]. Hermann et al. [18] used a combination of process and outcome indicators e.g. case management and length of stay; and Chung et al. [17] used structure, process and outcome indicators. One study [20] used a mixture of process and outcome indicators, while another study [25] used a combination of structural and process indicators. Most studies used quantitative indicators, such as 5-year over-all survival rate [17]. Roberts et al. [28] describe the use of qualitative and quantitative indicators.

### PB outcomes

Looking at the outcomes of the different pathway studies it can be seen that these cover a wide range of topics, Brucker [27] for example provided proof of concept for the feasibility of a nationwide system for benchmarking. The goal of establishing a nationwide network of certified breast centres in Germany can be considered largely achieved according to Wallwiener [25]. Wesselman [26] shows that most of the targets for indicators for colorectal care are being better met over the course of time.

Mainz et al. [19] reported a major difference between the Nordic countries with regard for 5 years survival for prostate cancer. However, they also reported difficulties such as: threats to comparability when comparing quality at the international level, this is mainly related to data collection. Stolar [22] showed that pediatric surgeons are unable to generate sufficient direct financial resources to support their employment and practice operational expenses. Outcomes of the other studies can be found in Table 3.

### PB Impact

One article identified improvements in the diagnosis of the patient and provision of care related to participating in the benchmark for example improvements in the preoperative histology and radiotherapy after mastectomy [27]. Three articles identified suggestions for improvements based on the benchmark [20, 22, 24], in the provision of care for instance on the use of opiates at the end of life [17] and improvements on the organizational level such as the decrease of the frequency of hospital visits, lead times and costs [22]. For other improvements see Table 3.

### PB Success factors

One study identified success factors. According to Brucker [27] a success factor within their project was the fact that participation was voluntary and all the data was handled anonymous.

### II Institutional benchmarking (IB)

A summary analysis of the institutional benchmarking studies can be found in Table 4.

**Table 4:** Summary of the analysis of the institutional benchmarking projects. N.A. = Not applicable

Author	Study design	Benchmarking model and/or steps	Indicators	Outcome	Impact (improvements/improvement suggestions)	Success factors
Barr [30]	Multi comparisons study using the National Practice Benchmark.	Partner: Industry Content: Performance/Strategic Purpose: Collaborative National Practice Benchmark survey	N.A.	The National Practice Benchmark reveals a process of change that is reasonably orderly and predictable, and demonstrates that the adaptation of the oncology community is directional, moving toward gains in efficiency as assessed by a variety of measures.	N.A.	To make the survey more accessible, it was stratified into 2 sections (minimum data set and extra).
Brann [31]	Multi comparisons study in which representatives from child and adolescent mental health organizations used eight benchmarking forums to compare performance against relevant KPIs.	N.A.	Key performance indicators looking at outcomes in mental health	Benchmarking has the potential to illuminate intra- and inter-organizational performance.	N.A.	1. Commitment of the management and securing resources. 2. Feeding back benchmarking data to data interpretation clinical staff to maintain their motivation to the project. 3. Forums for participants to provide them with the opportunity to discuss the performance of their organisation and draw lessons from other organisations.
De Korne [3]	Mixture of methods: a systematic literature review and semi-structured interviews. An evaluation frame (based on a systematic literature review) was applied longitudinally to a case study of nine eye hospitals that used a set of performance indicators for benchmarking.	Partner: Industry/Global Content: Process/Performance Purpose: Collaborative 4P model : 1) the purposes of benchmarking; 2) the performance indicators used; 3) the participating organizations; and 4) the organizations’ performance management systems.	Performance outcome indicators	The benchmarking indicators were mostly used to initiate and to facilitate discussions about management strategies. The eye hospitals in this study were not successful in reaching the goal of quantifying performance gaps or identifying best practices.	Indicators for benchmarking were not incorporated in a performance management system in any of the hospitals, nor were results discussed with or among employees; only the strategic level was involved.	Performance indicators should; 1. Represent strategically important items; 2.the indicators have to be specific, measurable, acceptable, achievable, realistic, relevant, and timely (SMART); 3. Data have to be converted into measurable quantities; 4. the indicator information has to be comparable to those of other organizations; 5. selected indicators must be relevant to the benchmarking purposes; 6. the indicators should have validity with respect to performance and participants and should also discriminate.
De Korne [25]	Mixture of methods: quantitative analysis included (i) analysis of fiscal year 2009 benchmarking performance data and (ii) evaluation of multiple cases by applying an evaluation frame abstracted from the literature to five U.S. eye hospitals that used a set of 10 indicators for efficiency benchmarking. Qualitative analysis of interviews, document analyses, and questionnaires.	Partner: Industry Content: Performance Purpose: Collaborative 4P model : 1) the purposes of benchmarking; 2) the performance indicators used; 3) the participating organizations; and 4) the organizations’ performance management systems.	Efficiency outcome indicators	The benchmark initiative fulfilled many of its purposes, namely, identifying performance gaps, implementing best practices, and stimulating exchange of knowledge.	Case studies showed that, to realize long-term efforts, broader cooperation is necessary.	1. the 4P model suggests that reliable and comparable indicators are a precondition for a successful benchmark, 2. case studies suggest that the development process is an important part of benchmarking. 3. homogeneity in language, reimbursement systems, and administrations
Schwappach [26]	Prospective and retrospective mixed methods: Questionnaires, Demographic, clinical, and performance data collected via specific data sheets; systematic data controlling.	Partner: Industry Content: Process/Performance Purpose: Collaborative EMERGE: (1) selection of interested hospitals, participating on a voluntary basis; (2) joint development of a set of clinical performance indicators agreed upon by all parties; (3) establishment of a measurement system, development of measurement tools and design of data collection instruments; (4) data collection in a first measurement cycle; (5) benchmarking of results and definition of shared, quantitative targets; (5) initialization of hospital-specific improvement activities; (6) data collection in a second measurement cycle; and (7) benchmarking of results.	Outcome Indicator set including two main components: objective measures that evaluate clinical performance in terms of speed and accuracy of patient assessment, and patients’ experiences with care provided by Eds.	Concordance of prospective and retrospective assignments to one of three urgency categories improved significantly by 1%, and both under- and over-prioritization, were reduced. Significant improvements in the reports provided by patients were achieved and were mainly demonstrated in structures of care provision and perceived humanity.	A number of improvement activities were initiated in individual hospitals covering a wide range of targets, from investment in ED structures to professional education and organization of care.	Interpretation of results should be guided by a culture of organisational learning rather than individual blame.
Shaw [30]	Multi comparisons study with the use of questionnaire containing ten questions.	N.A.	10 ‘questions’ regarding ED patient utilization, wait times, services, and attending physician staffing of the nation’s PEDs. Indicators qualified as outcome indicators.	Benchmarking of PEM staffing and performance indicators by PEM directors yields important administrative data. PEDs have higher census and admission rates compared with information from all EDs, while their attending staffing, wait times, and rate of patients who leave without being seen are comparable to those of general EDs.	In larger departments, the opening of fast tracks during high census times has allowed for shorter disposition of lower acuity patients with good success, this has been recommended as one of the solutions to better ED throughput.	N.A.
Van Lent [6]	Multi comparisons study internationally benchmarking operations management in cancer centres.	Partner: Industry/Global Content: Performance Purpose: Collaborative. Spendolinis method and a new 13^d^ step: 1. Determine what to benchmark; 2. Form a benchmarking team; 3. Choose benchmarking partners; 4. Define and verify the main characteristics of the partners; 5. Identify stakeholders; 6. Construct a framework to structure the indicators; 7. Develop relevant and comparable indicators; 8. Stakeholders select indicators; 9. Measure the set of performance indicators; 10. Analyze performance differences; 11. Take action: results were presented in a report and recommendations were given; 12. Develop improvement plans; and 13. Implement the improvement plans	Outcome indicators containing a numerator and a de-numerator The selected indicators distinguished between the total organization level, diagnostics, surgery, medication related treatments, radiotherapy and research.	The results on the feasibility of benchmarking as a tool to improved hospital processes are mixed. Success factors identified are a well-defined and small project scope, partner selection based on clear criteria, stakeholder involvement, simple and well-structured indicators, and analysis of both the process and its results.	All multiple case studies provided areas for improvement and one case study presented the results of a successful improvement project based on international benchmarking.	1. Internal stakeholders must be convinced that others might have developed solutions for problems that can be translated to their own settings. 2. Management must reserve sufficient resources for the total benchmarks. 3. Limit the scope to a well-defined problem. 4. Define criteria to verify the comparability of benchmarking partners based on subjects and process. 5. Construct a format that enables a structured comparison. 6. Use both quantitative and qualitative data for measurement. 7. Involve stakeholders to gain consensus about the indicators.8. Keep indicators simple so that enough time can be spent on the analysis of the underlying processes. 9. For indicators showing a large annual variation in outcomes, measurement over a number of years should be considered. 10. Adapt the identified better working methods so that they comply with other practices in the organisation.

### IB Study design

In the two articles by de Korne [3, 29] mixed methods were used to develop an evaluation frame for benchmarking studies in eye-hospitals. Barr et al.[30] used the National Practice Benchmark to collect data on Oncology Practice Trends. Brann [31] developed forums for benchmarking child and youth mental-health. Van Lent et al.[6] conducted three independent international benchmarking studies on operations management of comprehensive cancer centers and chemotherapy day units. Schwappach [32] used a pre–post design in two measurement cycles, before and after implementation of improvement activities at emergency departments. Shaw [33] used a questionnaire with 10 questions to collect data on pediatric emergency departments. More information on study design can be found in Table 4.

### IB Benchmark model

Characterizing the benchmark models and/or steps with the scheme by Fong [16] it can be seen that all studies used partners from the industry, in two studies these partners were global. Two articles benchmarked performance [6, 29] while two other articles benchmarked both processes as performance [3, 32] and one article reported the benchmarking of performance and strategies [30]. More detailed information on the benchmark models can be found in Table 4.

### IB Indicators

Most of the studies used outcome indicators (*N* = 6) [3, 6, 29, 31–33]. Schwappach et al. [32] for example used indicators to evaluate speed and accuracy of patient assessment, and patients’ experiences with care by emergency departments. Van Lent [6] described the use of indicators that differentiated between the organizational divisions of cancer centers such as diagnostics, radiotherapy and research. Brann [31] used Key Performance Indicators such as 28-day readmissions to inpatient settings, and cost per 3-month community care period.

### IB Outcomes

Different outcomes were mentioned in the study by de Korne [3] and on different aspects of operations management by van Lent [6]. However van Lent also showed that the results on the feasibility of benchmarking as a tool to improve hospital processes are mixed. The National Practice Benchmark (NPB) [30] demonstrated that the adaptation of oncology practices is moving toward gains in efficiency. Outcomes of the study by Schwappach [32] showed that improvements in the reports provided by patients were mainly demonstrated in structures of care provision and perceived humanity. Shaw [33] showed that benchmarking of staffing and performance indicators by directors yields important administrative data. Brann et al. [31] presented that benchmarking has the potential to illuminate intra- and inter-organizational performance.

### IB Improvements

Improvements mentioned due to participating in the benchmark (Table 4) were a successful improvement project [6] leading to a 24% increase in bed utilization and a 12% increase in productivity in cancer centers and investments in Emergency Department (ED) structures, professional education and improvement of the organization of care [29].

### IB Success factors

Almost all institutional benchmarking articles identified success factors (*N* = 7). Frequently mentioned factors were commitment of management [6, 31] and the development of good indicators [3, 6, 29].

### III Benchmarking evaluation/methodology (BEM)

A summary analysis of the benchmarking evaluation/methodology studies can be found in Table 5.

**Table 5:** Summary of the analysis of the benchmarking evaluation/methodology studies

Author	Study design	Benchmarking model and/or steps	Indicators	Outcome	Impact (improvements/improvement suggestions)	Success factors
Ellershaw [34]	Survey to assess the usefulness of benchmarking with the Liverpool Care Pathway	Partner: Industry Content: Process Purpose: Collaborative	N.A.	Whilst almost three quarters of the respondents in the hospital sector felt that participation in the benchmark had had a direct impact on the delivery of care, only around a third in the other two sectors felt the same (hospice and community).	Specific improvements in levels of communication between health professionals and relatives, within multidisciplinary teams and across sectors occurred as a result of participation in the benchmarking exercise.	Holding a workshop for participants to reflect on data, enhances understanding and learn from others.
Ellis [35]	Literature review to encourage the acceptance and use of Essence of Care as a new benchmarking approach.	Partner: Industry Content: Process Purpose: CollaborativeEvaluation of a benchmark with the use of Essence of Care	N.A.	Essence of Care benchmarking is a sophisticated clinical practice benchmarking approach which needs to be accepted as an integral part of health service benchmarking activity to support improvement in the quality of patient care and experiences.	N.A.	1. Reciprocity
Matykiewicz [36]	Case study approach and qualitative methods namely interviews and focus groups	Partner: Industry Content: Process Purpose: Collaborative The Essence of Care process includes: 1) Agree best practice; 2) Assess clinical areas against best practice; 3) Produce/implement action plan aimed at achieving best practice; 4) Review achievement of best practice; 5) Disseminate improvement and/or review action plan; 6) Agree best practice.	Best practice indicators	Whilst raising awareness is relatively straightforward, putting Essence of Care into practice is more difficult to achieve, especially when happening at a time of significant organizational change.	Through self-assessment against the best practice indicators, a problem was identified which, if not dealt with, could have escalated to a more serious situation. The manager saw this as an opportunity to learn from mistakes and initiated a service review that has since resulted in the service being redesigned.	1. Workshops (successful in raising awareness, help people to understand how to apply the benchmarking process in practice)
Profit [37]	Literature review on composite indicator development, health systems, and quality measurement in the pediatric healthcare setting.	N.A.	No indicators were mentioned, however a conceptual framework to develop comprehensive, robust, and transparent composite indicators of pediatric care quality was developed. The model proposed identifying structural, process, and outcome metrics for each of the Institute of Medicine’s six domains of quality.	The combination of performance metric development methodology with Profit et al.’s quality matrix framework may result in a unique approach for quality measurement that is fair, scientifically sound, and promotes the all-important provider buy-in. The framework presented offers researchers a path to composite indicator development.	N.A.	N.A.

*N.A*. not applicable

### BEM Study design

Ellershaw [34] assessed the usefulness of benchmarking using the Liverpool Care Pathway in acute hospitals in England with the use of a questionnaire. Ellis [35] performed a review of benchmarking literature. Matykiewicz [36] evaluated the Essence of Care as a benchmarking tool with a case study approach and qualitative methods.

Profit [37] used a review of the scientific literature on composite indicator development, health systems, and quality measurement in pediatric healthcare. More information on study design can be found in Table 5.

### BEM Benchmark model/steps

Three studies describe a benchmark model. They all describe industry partners and process benchmarking (see Table 5).

### BEM Indicators

One article described the use of indicators, though very minimally. Matykiewicz [36] describes benchmarking against best practice indicators, but specific indicators are not mentioned. Profit et al. [37] developed a model for the development of indicators of quality of care.

### BEM Outcomes

The study by Ellershaw [34] displayed that almost three quarters of respondents in the hospital sector felt that participation in the benchmark had had a direct impact on the delivery of care. The outcomes of the study by Ellis [35] was that Essence of Care benchmarking is a sophisticated clinical practice benchmarking approach which needs to be accepted as an integral part of health service benchmarking activity. Matykiewicz [36] showed that whilst raising awareness is relatively straightforward, putting Essence of Care into practice is more difficult. Profit et al. [37] concluded that the framework they presented offers researchers an explicit path to composite indicator development.

### BEM Improvements

Improvements due to the benchmark exercise that were identified included specific improvements in levels of communication between health professionals and relatives, within multidisciplinary teams and across sectors [34] and that through self-assessment against best practice problems could be identified and solved [36].

### BEM Success factors

Three articles mentioned success factors, both Ellershaw [34] and Matykiewicz [36] mentioned the organization of a workshop, while Ellis [35] identified reciprocity as an important factor for success.

### IV Benchmark using patient registry data

The only benchmark study [38] using patient registry data originated in oncology practice in the US (see Table 6). For this study National Cancer Database (NCDB) reports from the Electronic Quality Improvement Packet (e-QUIP) were reviewed ensuring all network facilities are in compliance with specific outcome benchmarks. Outcome indicators such as local adherence to standard-of-care guidelines were used. A review of the e-QUIP-breast study at Carolinas Medical Center (CMC) showed that treatment methods could be improved. No improvements were reported. At CMC, the registry has been a key instrument in program improvement in meeting standards in the care of breast and colon cancer by benchmarking against state and national registry data.

**Table 6:** Summary of the analysis of Benchmark study using patient registry data

Author	Study design	Benchmarking model and/or steps	Indicators	Outcome	Impact (improvements/improvement suggestions)	Success factors
Greene [38]	Development of a cancer committee; review of the NCDB reports from the Electronic Quality Improvement Packet (e-QUIP) and CP3R ensuring all network facilities are in compliance with specific outcome benchmarks.	N.A.	Outcome indicators	In addition to a role in benchmarking, registry data may be used to assist in establishing new research protocols and in determining market share by the hospital administration. The registry identified several issues which included the lack of physician office contact information, and time lapse for treatment completion.	Two potential issues were identified. With instruction for the pathologists and surgeons regarding these issues, this rate is expected to improve.	N.A.

*N.A*. not applicable

## Discussion

There is a growing need for healthcare providers to focus on performance. Benchmarking is a common and supposedly effective method for measuring and analyzing performance [2]. Benchmarking in specialty hospitals developed from the quantitative measurement of performance to the qualitative measurement and achievement of best practice [39].

In order to inform the development of benchmark tool for comprehensive cancer care (the BENCH-CAN project) we assessed the study characteristics of benchmarking projects in specialty hospitals, avoid duplication and identified the success factors to benchmarking of specialty hospitals. This scoping review identified 24 papers that met the selection criteria which were allocated to one of four categories. Regarding our first two research objectives: (i) provide an overview of research on benchmarking in specialty hospitals and care pathways, (ii) describe study characteristics such as method, setting, models/frameworks, and outcomes, we reviewed the first three categories against a common set of five issues that shape the following discussion. The fourth category (*Benchmark using patient registry data*) had only a single paper so could not be appraised in the same way.

### I Area of practice

In terms of study settings, we were interested in the areas where benchmarking would be most frequently used. Our review identified seven types of specialty hospitals. Most studies were set in oncology specialty hospitals. The majority (*n* = 12) of the articles described projects in which part of a specialty hospital or care pathway was benchmarked. This could be due to the fact that one of the success factors of a benchmarking project defined by van Lent et al. [6] is the development of a manageable-sized project scope. This can be an identified problem in a department or unit (part of a specialty hospital), or a small process that involves several departments (care pathway).

### II Study design

Looking at the different study designs both quantitative as qualitative methods can be found. All institutional articles except Schwappach [29] (retrospective and prospective) made use of a prospective research design while most pathway articles used a retrospective multi-comparison design. Stakeholders often played an important role in the benchmarking process and consensus methods such as the Delphi method were frequently used to develop the benchmarking indicators.

### III Benchmark model

Fifteen articles described a benchmark model/steps. All studies that described a benchmarking study made use of partners from the industry, in 4 articles these where from different countries, e.g. global. Most benchmarks were on performance (*N* = 8), others used a combination of performance and process benchmarking (*N* = 3) or performance and strategic benchmarking (*N* = 1). Three studies described a process benchmark and one benchmarking on strategies. The classification scheme was not developed for healthcare benchmarking specifically. This is shown by the definition of competitor. Some of the described partners in the benchmarking studies fit the first part of the definition: In business, a company in the same industry or a similar industry which offers a similar product or service [40] for example breast cancer centers or eye hospitals. However there is not always competition between these centers (second part definition). A healthcare specific scheme for benchmarking models would be preferred, this was however not found.

In some cases, a model has been uniquely developed–possibly using field expertise- for performing a particular type of benchmarking, which means that there was no evidence of the usability of the model beforehand. In their article on ‘Benchmarking the benchmarking models’ Anand and Kodali [15] however identify and recommend some common features of benchmarking models. Their cursory review of different benchmarking process models revealed that the most common steps are: “identify the benchmarking subject” and “identify benchmarking partners” [15]. The purpose of the benchmarking process models should be to describe the steps that should be carried out while performing benchmarking. Anand and Kodali [15] recommend that a benchmark model should be clear and basic, emphasizing logical planning and organization and establishing a protocol of behaviors and outcomes. Looking at the models described in this review it shows that only 5 articles describe models that have all the features described by Anand and Kodali [3, 6, 29, 32, 36].

### IV Registry

The article about the use of a registry differed in the sense that no benchmark model or benchmarking steps were described. Instead it focused on the usefulness of using a registry for benchmarking. According to Greene et al. [38] a registry is a valuable tool for evaluating quality benchmarks in cancer care. Sousa et al. [41] showed the general demands for accountability, transparency and quality improvement make the wider development, implementation and use of national quality registries for benchmarking, inevitable. Based on this we had expected to find more articles describing the use of the registry for benchmarking, these were however not identified through our search.

### V Indicators

Currently, it seems that the development of indicators for benchmarking is the main focus of most benchmarking studies. The importance of indicator development is highlighted by Groene et al. [42] who identified 11 national indicator development projects. Papers included in this study showed a wide array of approaches to define and select indicators to be used in the projects, such as interviews, focus groups, literature reviews and consensus surveys (Delphi method and others).

A review by Nolte [43] shows that there is an ongoing debate about the usefulness of process versus outcome indicators to evaluate healthcare quality. In most papers included in this study outcome indicators were used, especially in the pathway benchmarking papers. This seems contradictory to findings by Mant [44] who noted that the relevance of outcome measures is likely to increase towards macro-level assessments of quality, while at the organizational or team level, process measures will become more useful. Based on this one would expect the use of process indicators for especially the pathway articles.

### Benchmarking as a tool for quality improvement and success factors

Regarding our third objective: “verify the quality of benchmarking as a tool to improve quality in specialty hospitals and identify success factors” we found the following. Only six articles described improvements related to the benchmark. Specific improvements were described in the level of communication between health professionals and relatives, within multidisciplinary teams and across sectors; service delivery and organization of care; and pathway development. Only three articles actually showed the improvement effects of doing a benchmark in practice. This could be linked to the fact that almost no benchmark model described a last step of evaluation of improvement plans as being part of the benchmark process. Brucker [27] showed that nationwide external benchmarking of breast cancer care is feasible and successful. Van Lent [6] however showed that the results on the feasibility of benchmarking as a tool to improved hospital processes were mixed. This makes it difficult to assess whether benchmarking is a useful tool for quality improvement in specialty hospitals.

Within the pathway studies only one paper mentioned success factors, in contrast with almost all institutional and benchmark evaluation- and methodology papers. Based on our review we’ve come up with a list of success factors for benchmarking specialty hospitals or care pathways (Table 7). One article exploring the benchmarking of Comprehensive Cancer Centres [6] produced a detailed list of success factors for benchmarking project (see Table 7), such as a well-defined and small project scope and partner selection based on clear criteria. This might be easier for specialty hospitals due to the specific focus and characteristics than for general hospitals. Organizing a meeting for participants, either before or after the audit visits, was mentioned as a success factor [34, 36]. Those workshops or forums provided the opportunity for participants to network with other organizations, discuss the meaning of data and share ideas for quality improvements and best practices. Especially the development of indicators was mentioned often, corresponding to our earlier observation about the emphasis that is put on this issue.

**Table 7:** Success factors benchmarking projects specialty hospitals and pathways

1. Voluntary participation
2. Anonymous participation
3. Internal stakeholders must be convinced that others might have developed solutions for problems of the underlying processes that can be translated to their own settings.
4. Verify homogeneity participant group to ensure the comparability of benchmarking partners
5. Ensure commitment of the management and secure resources
6. Limit the scope of the project to a well-defined problem
7. Involve stakeholders to gain consensus about the indicators
8. Develop indicators that are specific, measurable, acceptable, achievable, realistic, relevant, and timely (SMART)
9. Use simple indicators so that enough time can be spent on the analysis
10. Measure both qualitative and quantitative data
11. Stratify survey into minimum data set and additional extra’s
12. For indicators showing a large annual variation in outcomes, measurement over a number of years should be considered
13. Feed benchmarking data back to clinical staff to maintain their motivation to the project
14. Organize forums and workshops for participants to discuss performance of their organization and learn from other organizations
15. Convert data into measurable quantities
16. Homogeneity in language, reimbursement systems, and administrations
17. Interpretation of results should be guided by a culture of organisational learning rather than individual blame.

Although this scoping review shows that the included studies seem to focus on indicator development rather than the implementation and evaluation of benchmarking, the characteristics described (especially the models) can be used as a basis for future research. Researchers, policy makers or other actors that wish to develop benchmarking projects for specialty hospitals should learn lessons from previous projects to prevent the reinvention of the wheel. The studies in this review showed that ensuring the commitment to the project by the management team of hospitals participating and the allocation of sufficient resources for the completion of the project is paramount to the development of a benchmarking exercise. The information found in combination with the provided success factors may increase the chance that benchmarking results in improved performance in specialty hospitals like cancer centers in the future.

### Limitations

A potential limitation is that by searching the titles and abstracts we may have missed relevant papers. The articles included in this review were not appraised for their scientific rigor, as scoping reviews do not typically include critical appraisals of the evidence. In deciding to summarize and report the overall findings without the scrutiny of a formal appraisal, we recognize that our results speak to the extent of the setting and model of the benchmark study rather than provide the reader with support for the effectiveness of benchmarking.

## Conclusion

Benchmarking in specialty hospitals developed from simple data comparison to quantitative measurement of performance, qualitative measurement and achievement of best practice. Based on this review it seems however that benchmarking in specialty hospitals is still in development. Benchmarking seems to be most reported up on and possibly developed in the field of oncology and eye hospitals, however most studies do not describe a structured benchmarking method or a model that can be used repeatable. Based on our study we identified a list of success factors for benchmarking specialty hospitals. Developing ‘good’ indicators was mentioned frequently as a success factor. Within the included papers there seems to be a focus on indicator development rather than measuring performances, which is an indication of development rather than implementation. Further research is needed to ensure that benchmarking in specialty hospitals fulfills its objective, to improve the performance of healthcare facilities. Researchers wishing –as a next step- to evaluate the effectiveness of benchmarking to improve quality in specialty hospitals, should conduct evaluations using robust and structured designs, focusing on outcomes of the benchmark and preferably do a follow up to check whether improvement plans were implemented.

## Additional file

Additional file 1: Full search strategies PubMed and EMBASE (DOC 107 kb)

## Abbreviations

BC: Breast cancer
BEM: Benchmarking evaluation/methodology
CCC: Comprehensive cancer center
CMC: Carolinas medical center
DKG: The German cancer society
ED: Emergency department
e-QUIP: Electronic quality improvement packet
IB: Institutional benchmarking
KPI: Key performance indicators
N.A.: Not applicable
NCCN: National comprehensive cancer network
NCDB: National cancer database
NHS: National health system
NPB: National practice Benchmark
PB: Pathway benchmarking
PED: Pediatric emergency department
QI: Quality indicator
SMART: Specific, Measurable, Achievable, Realistic, Relevant, Timely
UK: United Kingdom
USA: United States of America
