# Supplementary material for: Benchmarking specialty hospitals, a scoping review on theory and practice
Source: BMC Health Serv Res. 2017 Apr 4;17:245. doi: 10.1186/s12913-017-2154-y (PMC5379508; doi:10.1186/s12913-017-2154-y)
Supplement: Additional file 1: — Full search strategies PubMed and EMBASE (DOC 107 kb) [file 12913_2017_2154_MOESM1_ESM.doc]

**ANNEX 1**

**First Search Pubmed**

| Search | Query | Items found |
| --- | --- | --- |
| #22 | Search #16 AND #17 AND organization* [tiab] Filters: Publication date from 2006/01/01 to 2014/12/31; English | **176** |
| #21 | Search #16 AND #17 AND organization* [tiab] Filters: Publication date from 2006/01/01 to 2014/12/31 | 189 |
| #20 | Search #16 AND #17 AND organization* [tiab] Filters: Publication date from 1996/01/01 to 2014/12/31; English | 261 |
| #19 | Search #16 AND #17 AND organization* [tiab] Filters: Publication date from 1996/01/01 to 2014/12/31 | 289 |
| #17 | Search #14 OR #15 | 113084 |
| #16 | Search #12 OR #13 | 174389 |
| #15 | Search special hospital* [tiab] OR ((cancer care [tiab] OR cardiac care [tiab]) AND (facilit* [tiab] OR hospital* [tiab])) OR hospice* [tiab] OR ((chronic disease [tiab] OR oncology [tiab] convalescent [tiab] OR isolation [tiab] OR maternity [tiab] OR osteopathic [tiab] OR pediatric [tiab] OR psychiatric [tiab]) AND (hospital* [tiab])) OR surgicenter* [tiab] | 75326 |
| #14 | Search special hospitals [mesh] | 50902 |
| #13 | Search benchmarking [tiab] OR best practice* [tiab] OR ((quality [tiab]) AND (improvement* [tiab] OR assurance* [tiab] OR control [tiab])) | 173525 |
| #12 | Search benchmarking [mesh] OR quality improvement [mesh] OR quality of health care [mesh] OR quality assurance, health care [mesh] OR quality control [mesh] OR outcome and process assessment (health care) [mesh] | 945 |

**First search EMBASE**

| Query | Items found |
| --- | --- |
| 'benchmarking'/exp OR benchmarking OR benchmark OR best AND practice AND [2003-2014]/py OR (benchmarking:ab,ti OR benchmark:ab,ti OR best AND practice:ab,ti) AND (special AND hospitals OR 'specialty hospital':ab,ti) | 168 |
| special AND hospitals OR 'specialty hospital':ab,ti | 6793 |
| 'benchmarking'/exp OR benchmarking OR benchmark OR best AND practice AND [2003-2014]/py OR (benchmarking:ab,ti OR benchmark:ab,ti OR best AND practice:ab,ti) | 110896 |
| 'specialty hospital':ab,ti | 353 |
| special AND hospitals | 6442 |
| benchmarking:ab,ti OR benchmark:ab,ti OR best AND practice:ab,ti | 32652 |
| 'benchmarking'/exp OR benchmarking OR benchmark OR best AND practice AND [2003-2014]/py | 103509 |

**Second search Pubmed**

| Search | Query | Items found |
| --- | --- | --- |
| #45 | Search(#42) AND #43Filters: Publication date from 2003/01/01 to 2014/12/31 | **196** |
| #43 | Search project* [tiab] | 217693 |
| #42 | Search ((#39) AND #40) AND #41 | 4071 |
| #41 | Search (#38) OR #30 | 121675 |
| #40 | Search (#36) OR #37 | 424176 |
| #39 | Search (organization and administration [mesh]) | 1038696 |
| #30 | Search special hospitals [mesh] | 51090 |
| #38 | Search(special hospital* [tiab] OR specialty hospital* [tiab] OR specialized hospital* OR ((cancer care [tiab] OR cardiac care [tiab]) AND (facilit* [tiab] OR hospital* [tiab])) OR hospice* [tiab] OR ((chronic disease [tiab] OR oncology [tiab] OR convalescent [tiab] OR isolation [tiab] OR maternity [tiab] OR osteopathic [tiab] OR pediatric [tiab] OR psychiatric [tiab]) AND (hospital* [tiab])) OR surgicenter* [tiab]) | 84380 |
| #37 | Search (benchmarking [tiab] OR best practice* [tiab] OR ((quality [tiab]) AND (improvement* [tiab] OR assurance* [tiab] OR control [tiab]))) | 175782 |
| #36 | Search (benchmarking [mesh] OR quality improvement [mesh] OR quality assurance, health care [mesh] OR quality control [mesh]) | 288665 |

**Second search EMBASE**

| Query | Items found |
| --- | --- |
| special AND hospitals AND organization AND administration AND ('benchmarking'/exp OR benchmarking OR quality AND improvement OR quality AND assurance, AND ('health'/exp OR health) AND care OR quality AND ('control'/exp OR control) AND [2006-2014]/py OR ('benchmarking':ab,ti OR best AND practice*:ab,ti) OR (quality:ab,ti AND (improvement*:ab,ti OR assurance*:ab,ti OR control:ab,ti))) | **67** |
| 'benchmarking'/exp OR benchmarking OR quality AND improvement OR quality AND assurance, AND ('health'/exp OR health) AND care OR quality AND ('control'/exp OR control) AND [2006-2014]/py OR ('benchmarking':ab,ti OR best AND practice*:ab,ti) OR (quality:ab,ti AND (improvement*:ab,ti OR assurance*:ab,ti OR control:ab,ti)) | 1090705 |
| organization AND administration | 245778 |
| special AND hospitals | 6442 |
| 'benchmarking':ab,ti OR best AND practice*:ab,ti OR (quality:ab,ti AND (improvement*:ab,ti OR assurance*:ab,ti OR control:ab,ti)) | 258785 |
| 'benchmarking'/exp OR quality AND improvement OR quality AND assurance, AND 'health'/exp AND care OR quality AND 'control'/exp AND [2006-2014]/py | 153474 |

**Third search Pubmed**

| Search | Query | Items found |
| --- | --- | --- |
| #10 | Search (#7) AND #8 Filters: Publication date from 2006/01/01 to 2014/12/31; English | **261** |
| #9 | Search (#7) AND #8 Filters: English | 444 |
| #8 | Search (project*[tiab]) | 222793 |
| #7 | Search (#5) AND #6 | 6358 |
| #6 | Search (#3) OR #4 | 124155 |
| #5 | Search (#1) OR #2 | 274481 |
| #4 | Search (special hospitals[mesh]) | 51583 |
| #3 | Search ((special hospital* [tiab] OR specialty hospital* [tiab] OR specialized hospital* OR ((cancer care [tiab] OR cardiac care [tiab]) AND (facilit* [tiab] OR hospital* [tiab])) OR hospice* [tiab] OR ((chronic disease [tiab] OR oncology [tiab] OR convalescent [tiab] OR isolation [tiab] OR maternity [tiab] OR osteopathic [tiab] OR pediatric [tiab] OR psychiatric [tiab]) AND (hospital* [tiab])) OR surgicenter* [tiab])) | 86574 |
| #2 | Search ((benchmarking[tiab] OR best practice*[tiab] OR ((quality[tiab]) AND (improvement*[tiab] OR assurance*[tiab] OR control[tiab])) | 182296 |
| #1 | Search ((organization and administration[mesh]) AND ((benchmarking[mesh] OR quality improvement[mesh] OR quality assurance, health care[mesh] OR quality control[mesh])) | 109272 |

**Third search EMBASE**

| Query | Items found |
| --- | --- |
| ((('benchmarking'/syn AND [2003-2014]/py) AND (special AND hospitals)) NOT (clinical:ab,ti OR clinically:ab,ti OR therapeutic:ab,ti)) AND (2003:py OR 2004:py OR 2005:py OR 2006:py OR 2007:py OR 2008:py OR 2009:py OR 2010:py OR 2011:py OR 2012:py OR 2013:py) | 324 |
| (('benchmarking'/syn AND [2003-2014]/py) AND (special AND hospitals)) NOT (clinical:ab,ti OR clinically:ab,ti OR therapeutic:ab,ti) | 324 |
| ('benchmarking'/syn AND [2003-2014]/py) AND (special AND hospitals) | 532 |
| clinical:ab,ti OR clinically:ab,ti OR therapeutic:ab,ti | 3672600 |
| special AND hospitals | 6419 |
| 'benchmarking'/syn AND [2003-2014]/py | 880895 |

**Fourth search Pubmed**

| Search | Query | Items found |
| --- | --- | --- |
| #5 | Search (#4) NOT #3 Filters: Publication date from 2006/01/01 to 2014/12/31; English | **175** |
| #4 | Search (#1) AND #2 | [616](http://www.ncbi.nlm.nih.gov/pubmed/?cmd=HistorySearch&querykey=32) |
| #3 | Search (clinical[tiab] OR clinically[tiab] OR therapeutic[tiab]) | [2933746](http://www.ncbi.nlm.nih.gov/pubmed/?cmd=HistorySearch&querykey=27) |
| #2 | Search ((((special hospital* [tiab] OR specialty hospital* [tiab] OR specialized hospital* OR ((cancer care [tiab] OR cardiac care [tiab]) AND (facilit* [tiab] OR hospital* [tiab])) OR hospice* [tiab] OR ((chronic disease [tiab] OR oncology [tiab] OR convalescent [tiab] OR isolation [tiab] OR maternity [tiab] OR osteopathic [tiab] OR pediatric [tiab] OR psychiatric [tiab]) AND (hospital* [tiab])) OR surgicenter* [tiab])))) OR (special hospitals[mesh]) | 124155 |
| #1 | Search (((benchmarking[tiab] OR benchmark[tiab] OR best AND practice[tiab])) | 25342 |

**Fourth search EMBASE**

| Search | Query | Items found |
| --- | --- | --- |
| # 9 | ((('benchmarking' OR 'benchmarking'/exp OR benchmarking OR benchmark OR healthcare AND benchmarking) OR (benchmarking:ab,ti OR benchmark:ab,ti OR healthcare AND 'benchmarking':ab,ti)) AND ((special AND hospital) OR (special AND hospital:ab,ti OR(cancer AND care OR cardiac AND care:ab,ti) AND ('facility':ab,ti OR hospital:ab,ti) OR hospice:ab,ti OR (chronic AND disease:ab,ti) OR oncology:ab,ti OR convalescent:ab,ti OR isolation:ab,ti OR maternity:ab,ti OR osteopathic:ab,ti OR pediatric:ab,ti OR psychiatric:ab,ti AND 'hospital':ab,ti OR 'surgicenters':ab,ti))) AND (2003:py OR 2004:py OR 2005:py OR 2006:py OR 2007:py OR 2008:py OR 2009:py OR 2010:py OR 2011:py OR 2012:py OR 2013:py) | **135** |
| # 8 | (('benchmarking' OR 'benchmarking'/exp OR benchmarking OR benchmark OR healthcare AND benchmarking) OR (benchmarking:ab,ti OR benchmark:ab,ti OR healthcare AND 'benchmarking':ab,ti)) AND ((special AND hospital) OR (special AND hospital:ab,ti OR (cancer AND care OR cardiac AND care:ab,ti) AND ('facility':ab,ti OR hospital:ab,ti) OR hospice:ab,ti OR (chronic AND disease:ab,ti) OR oncology:ab,ti OR convalescent:ab,ti OR isolation:ab,ti OR maternity:ab,ti OR osteopathic:ab,ti OR pediatric:ab,ti OR psychiatric:ab,ti AND 'hospital':ab,ti OR 'surgicenters':ab,ti)) | 278 |
| # 7 | (special AND hospital) OR (special AND hospital:ab,ti OR (cancer AND care OR cardiac AND care:ab,ti) AND ('facility':ab,ti OR hospital:ab,ti) OR hospice:ab,ti OR (chronic AND disease:ab,ti) OR oncology:ab,ti OR convalescent:ab,ti OR isolation:ab,ti OR maternity:ab,ti OR osteopathic:ab,ti OR pediatric:ab,ti OR psychiatric:ab,ti AND 'hospital':ab,ti OR 'surgicenters':ab,ti) | 212,756 |
| # 6 | special AND hospital:ab,ti OR (cancer AND care OR cardiac AND care:ab,ti) AND ('facility':ab,ti OR hospital:ab,ti) OR hospice:ab,ti OR (chronic AND disease:ab,ti) OR oncology:ab,ti OR convalescent:ab,ti OR isolation:ab,ti OR maternity:ab,ti OR osteopathic:ab,ti OR pediatric:ab,ti OR psychiatric:ab,ti AND 'hospital':ab,ti OR 'surgicenters':ab,ti | 160,683 |
| # 5 | special AND hospital | 70,755 |
| # 4 | ('benchmarking' OR 'benchmarking'/exp OR benchmarking OR benchmark OR healthcare AND benchmarking) OR (benchmarking:ab,ti OR benchmark:ab,ti OR healthcare AND 'benchmarking':ab,ti) | 5,350 |
| # 3 | benchmarking:ab,ti OR benchmark:ab,ti OR healthcare AND 'benchmarking':ab,ti | 4,969 |
| # 2 | 'benchmarking' OR 'benchmarking'/exp OR benchmarking OR benchmark OR healthcare AND benchmarking | 5,350 |
| # 1 | 'benchmarking' OR 'benchmarking'/exp OR benchmarking OR benchmark | 282,781 |

**Fifth search Pubmed**

| Search | Query | Items found |
| --- | --- | --- |
| [#20](http://www.ncbi.nlm.nih.gov/pubmed/advanced) | Search (#18) AND #12 | **3****15** |
| [#18](http://www.ncbi.nlm.nih.gov/pubmed/advanced) | Search (#15) OR #13 | [39182](http://www.ncbi.nlm.nih.gov/pubmed/?cmd=HistorySearch&querykey=18) |
| [#17](http://www.ncbi.nlm.nih.gov/pubmed/advanced) | Search (#15) OR #13 Filters: Abstract; Publication date from 2003/01/01 to 2014/02/15; English | [39182](http://www.ncbi.nlm.nih.gov/pubmed/?cmd=HistorySearch&querykey=17) |
| [#16](http://www.ncbi.nlm.nih.gov/pubmed/advanced) | Search (special hospital* [tiab] OR ((cancer care [tiab] OR cardiac care [tiab]) AND (facilit* [tiab] OR hospital* [tiab])) OR hospice* [tiab] OR ((chronic disease [tiab] OR oncology [tiab] convalescent [tiab] OR isolation [tiab] OR maternity [tiab] OR osteopathic [tiab] OR pediatric [tiab] OR psychiatric [tiab]) AND (hospital* [tiab])) OR surgicenter* [tiab]) | [80232](http://www.ncbi.nlm.nih.gov/pubmed/?cmd=HistorySearch&querykey=16) |
| [#15](http://www.ncbi.nlm.nih.gov/pubmed/advanced) | Search (special hospital* [tiab] OR ((cancer care [tiab] OR cardiac care [tiab]) AND (facilit* [tiab] OR hospital* [tiab])) OR hospice* [tiab] OR ((chronic disease [tiab] OR oncology [tiab] convalescent [tiab] OR isolation [tiab] OR maternity [tiab] OR osteopathic [tiab] OR pediatric [tiab] OR psychiatric [tiab]) AND (hospital* [tiab])) OR surgicenter* [tiab]) Filters: Abstract; Publication date from 2003/01/01 to 2014/02/15; English | [33624](http://www.ncbi.nlm.nih.gov/pubmed/?cmd=HistorySearch&querykey=15) |
| [#14](http://www.ncbi.nlm.nih.gov/pubmed/advanced) | Search special hospitals [mesh] | [52244](http://www.ncbi.nlm.nih.gov/pubmed/?cmd=HistorySearch&querykey=14) |
| [#13](http://www.ncbi.nlm.nih.gov/pubmed/advanced) | Search special hospitals [mesh] Filters:Abstract; Publication date from 2003/01/01 to 2014/02/15; English | [9508](http://www.ncbi.nlm.nih.gov/pubmed/?cmd=HistorySearch&querykey=13) |
| [#12](http://www.ncbi.nlm.nih.gov/pubmed/advanced) | Search (#5) OR #7 | [17318](http://www.ncbi.nlm.nih.gov/pubmed/?cmd=HistorySearch&querykey=12) |
| [#11](http://www.ncbi.nlm.nih.gov/pubmed/advanced) | Search (#5) OR #7 Filters: Abstract; Publication date from 2003/01/01 to 2014/02/15; English | [17318](http://www.ncbi.nlm.nih.gov/pubmed/?cmd=HistorySearch&querykey=11) |
| [#10](http://www.ncbi.nlm.nih.gov/pubmed/advanced) | Search (#7) OR #8 | 19696 |
| [#8](http://www.ncbi.nlm.nih.gov/pubmed/advanced) | Search benchmark*[Title/Abstract] | 19696 |
| [#7](http://www.ncbi.nlm.nih.gov/pubmed/advanced) | Search benchmark*[Title/Abstract] Filters: Abstract; Publication date from 2003/01/01 to 2014/02/15; English | 14290 |
| [#6](http://www.ncbi.nlm.nih.gov/pubmed/advanced) | Search (Healthcare Benchmarking) AND "Benchmarking"[Mesh] | 10279 |
| [#5](http://www.ncbi.nlm.nih.gov/pubmed/advanced) | Search (Healthcare Benchmarking) AND "Benchmarking"[Mesh]Filters:Abstract; | 4912 |
| [#2](http://www.ncbi.nlm.nih.gov/pubmed/advanced) | Search [benchmark*] | 27838 |
| [#1](http://www.ncbi.nlm.nih.gov/pubmed/advanced) | Search [benchmark*] Filters: Abstract; Publication date from 2003/01/01 to 2014/02/15; English | 1777 |
